# Less Known Gastrointestinal Manifestations of Drug Reaction with Eosinophilia and Systemic Symptoms (DRESS) Syndrome: A Systematic Review of the Literature

**DOI:** 10.3390/jcm10184287

**Published:** 2021-09-21

**Authors:** Djordje Jevtic, Igor Dumic, Terri Nordin, Amteshwar Singh, Nadezda Sulovic, Milan Radovanovic, Mladen Jecmenica, Tamara Milovanovic

**Affiliations:** 1 School of Medicine, University of Belgrade, 11000 Belgrade, Serbia; djordje965@gmail.com (D.J.); nsulovic@yahoo.com (N.S.); tamara.alempijevic@med.bg.ac.rs (T.M.); 2 Mayo Clinic College of Medicine and Science, Rochester, MN 55905, USA; Nordin.Terri@mayo.edu (T.N.); Radovanovic.Milan@mayo.edu (M.R.); 3 Department of Hospital Medicine, Mayo Clinic Health System, Eau Claire, WI 54702, USA; 4 Department of Family Medicine, Mayo Clinic Health System, Eau Claire, WI 54702, USA; 5 Department of Hospital Medicine, Johns Hopkins University, Baltimore, MD 21205, USA; asingh42@jhmi.edu; 6 Department of Hospital Internal Medicine, Johns Hopkins University School of Medicine, Baltimore, MD 21205, USA; 7 Gastroenterology Fellowship Program, The Wright Center for Graduate Medical Education, Scranton, PA 18501, USA; giomla@gmail.com; 8 Department of Gastroenterology and Hepatology, Clinical Center of Serbia, 11000 Belgrade, Serbia

**Keywords:** drug reaction, DRESS syndrome, eosinophilia, esophagitis, colitis, pancreatitis, enteritis, diabetes mellitus

## Abstract

Drug reaction with eosinophilia and systemic symptoms (DRESS) syndrome is a potentially life threatening severe cutaneous drug reaction. Most patients develop eosinophilia, a rash, a fever, lymphadenopathy and variable visceral organ involvement 2–6 weeks following exposure to the inciting medication. Unlike other severe cutaneous drug reactions, internal organ involvement that leads to high mortality is a unique feature of DRESS syndrome. While the liver is the most common internal organ involved, literally every other visceral organ can be affected in this syndrome. The lesser-known gastrointestinal manifestations of this syndrome include esophagitis, gastritis, enteritis, colitis, pancreatitis and a late autoimmune sequela due to pancreatic injury such as fulminant type 1 diabetes mellitus, autoimmune type 1 diabetes mellitus and type 2 diabetes mellitus. While these entities are less common, they are associated with equally severe complications and adverse patient outcomes. In this review, we synthetize data on these rare manifestations using Preferred Reporting Items for Systematic Reviews and Meta-Analyses (PRISMA) guidelines. The liver, the most common visceral organ involved, has been described as part of DRESS elsewhere and is not included in the scope of this article.

## 1. Introduction

Drug reaction with eosinophilia and systemic symptoms (DRESS) syndrome (DS) is a rare, potentially life threatening severe cutaneous adverse reaction (SCAR). The main clinical features of DS are fever, eosinophilia, skin eruption, lymphadenopathy, or/and various degrees of visceral organ involvement [1].

Its pathophysiology includes a complex interaction between an individual’s genetic predisposition, alteration in drug metabolism and possible re-activation of latent viral infections [1]. Unlike other SCARs, the unique features of DRESS are a delayed onset of symptoms following exposure to the culprit medication (generally weeks rather than days), the unpredictable course, the involvement of various visceral organs and the delayed development of autoimmune sequelae [1,2]. However, a recent study raised a question that the delayed onset of DS following exposure to medication is not universally present and might depend on the class of medications [3].

To better categorize severe cutaneous drug reactions (Steven–Johnson syndrome, toxic epidermal necrolysis, acute generalized exanthematous pustulosis and DS), the RegiSCAR (European Registry of Severe Cutaneous Adverse Reactions) scoring system was developed [1,2]. Depending on the score, DS cases may be categorized as no case, possible case, probable case and definite case [1,2].

The syndrome’s nomenclature has significantly evolved since it was first described more than 80 years ago. Initially, it was named drug induced pseudo lymphoma (as lymphadenopathy, leukocytosis and fever mimicked lymphoma). Subsequently, the name changed to anticonvulsant hypersensitivity syndrome (as anticonvulsants are the most common culprit). Drug-induced hypersensitivity syndrome (DIHS) was the most recent term used before the current name DRESS was defined in 1996 by Bocquet et al. [1]. While the liver is the visceral organ most commonly involved, the syndrome can affect any internal organ including the heart, lungs, kidneys, pancreas, esophagus, stomach, small and large intestine and gallbladder. While the RegiSCAR scoring system lists the liver, kidneys, heart and pancreas specifically, other visceral organs’ involvement is labeled as “other” [1]. 

A late autoimmune sequela is a well-recognized feature of DS and include type 1 diabetes mellitus (T1DM), type 2 diabetes mellitus (T2DM), hypothyroidism, autoimmune hemolytic anemia and autoimmune enteropathy [4,5,6,7,8].

Gastrointestinal tract (GIT) involvement in DS is less common but also underreported [1,2,9,10,11,12]. The aim of this review is to describe rare GIT manifestations as part of DS, thereby raising awareness about these under recognized features of DS. The liver involvement, which is more common, has been described elsewhere [13] and is outside the scope of this review.

## 2. Materials and Methods

We performed systematic review of the literature by searching PubMed/Medline database for case reports and case series of DS associated with GIT involvement. GIT involvement was defined if the patients with DS had one or more of the following: esophagitis, gastritis, enteritic, colitis, pancreatitis and/or developed late autoimmune sequela due to pancreatic involvement such as various types of diabetes mellitus. All articles published from the inception of the database until September 2020 were analyzed. Review was conducted in accordance with PRISMA guidelines.

Search terms included “DRESS and esophagitis”, “DRESS and gastritis”, “DRESS and colitis”, “DRESS and pancreatitis”, “DIHS and esophagitis”, “DIHS and gastritis”, “DIHS and colitis” and “DIHS and pancreatitis”.

Two authors blindly and independently selected the cases. The two authors (D.J. and I.D.) agreed on article selection in 96% of cases. Remaining 4% of cases was resolved by consensus and in consultation with senior author (T.M.).

**Scheme 1:**
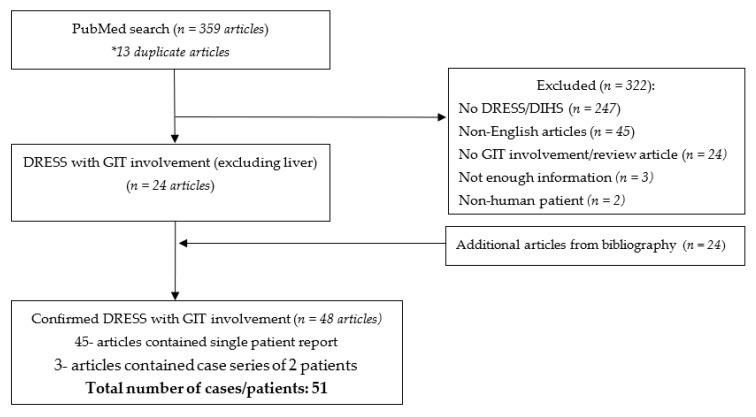
PRISMA flow chart.

Our search only included cases published in journals indexed by PubMed. The number of articles found on initial search was 359, of which 13 were duplicates. References of these articles were further searched, and 24 additional cases were identified. This resulted in total of 48 articles included in this review. Three of these articles reported case series of two patients, while the rest were singe case reports. Final number of cases included in this review is 51 [4,5,7,8,14,15,16,17,18,19,20,21,22,23,24,25,26,27,28,29,30,31,32,33,34,35,36,37,38,39,40,41,42,43,44,45,46,47,48,49,50,51,52,53,54,55,56,57]. PRISMA flow chart is illustrated in Scheme 1.

For cases reviewed, the RegiSCAR score was either reported initially by the authors or calculated based on availability of the information by authors. Only probable and definite cases were included. Cases that did not report enough information for RegiSCAR score to be calculated were included if clinical presentation, biopsy findings and/or treatment response were consistent with DS and all co-authors that selected the literature independently agreed.

An excel spreadsheet was created for tracking the following data: demographic characteristics (age, sex, race), comorbidities, precipitating drug, eosinophil count, visceral organ involvement, timing of GIT manifestation (latency), GIT endoscopy and biopsy, skin biopsy, presence of viral infection/reactivation and treatment and outcome of hospitalization. Latency was defined as the number of days from drug exposure to first GIT manifestation attributable to DS.

## 3. Results

### 3.1. Demographic Characteristics (Age, Sex, Race) and Co-Morbidities

The mean age of patients in this review was 41.6 years ± 3.1. The oldest patient was 80 years old and the youngest 9 months (0.75 years), nine were pediatric cases (17.6%) (<18 years of age). Twenty-seven patients (53%) were female. Only a minority of cases reported the patient’s race (19 of 51). Among those reported, most patients were of Asian descent (*n =* 8) (15.7%), followed by Caucasian (*n =* 3) (5.9%) and African American (*n =* 3) (5.9%). The most common comorbidities in this systematic review were epilepsy (*n =* 11, 21.6%), hypertension (*n =* 7, 13.7%) and gout (*n =* 5, 9.8%). The complete list of various comorbidities is presented in Table 1.

### 3.2. Medications, Eosinophilia and Latency

#### 3.2.1. Medications

The culprit drug was established in 45 patients (88.2%), with the most common being carbamazepine in 11 patients (21.6%). A complete list of the medications implicated as the cause of DS in this review is presented in Table 2.

#### 3.2.2. Eosinophilia

In this review, the median eosinophil count was 2530 cells/µL, ranging from 32 to 17,080 cells/µL. In our study, six patients (11.8%) did not demonstrate an increased AEC (defined by RegiSCAR as ≥700 or ≥10% if WBC < 4 × 10^9^). Furthermore, eosinophil count was not measured and/or reported in nine patients (17.6%).

#### 3.2.3. Latency

The mean latency in our review was 45.5 days ± 50.2. In 31 patients (60.1%), GIT manifestations were a part of the initial presentation or developed very shortly after hospital admission. In 17 patients (33.3%), GIT involvement occurred after significant time had passed from the onset of the first symptoms. The shortest observed latency in our review was two days in the patients treated with ciprofloxacin and allopurinol [22,44]. In this systematic review, we did not note any difference between the medication class and the latency period (53.1 days in antibiotic group versus 52.1 days in those who received anticonvulsants).

### 3.3. GIT Involvement

The symptoms of GIT involvement included diarrhea, vomiting, abdominal pain, nausea, epigastric pain and/or dysphagia (Table 3). The majority of reported cases included the pancreas (*n =* 29), (56.9%), followed by the large bowel (*n =* 21), (41.2%), esophagus (*n =* 4), small bowel (*n =* 3) and stomach (*n =* 1) (Table 4).

#### 3.3.1. Pancreas in DRESS Syndrome

**Figure 1:**
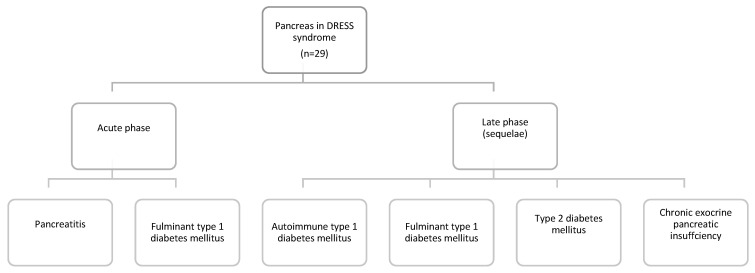
This figure summarizes types of pancreatic involvement.

Overall, the pancreas was the most common organ involved in 29 patients (56.9%). However, this includes the patients with acute pancreatitis and the patients who developed acute and late sequelae due to pancreatic involvement (Figure 1).

Pancreatic involvement as a part of DS may manifest either during an acute phase of the disease—acute pancreatitis and fulminant type 1 diabetes mellitus (T1DM), or as an acute sequela—autoimmune T1DM, fulminant T1DM, type 2 diabetes mellitus (T2DM) or chronic exocrine pancreatic insufficiency (Figure 1). The most common pancreatic manifestation of DS was T1DM (either as fulminant or autoimmune) in 18 patients (62.1%). As a sequela, T1DM (autoimmune or fulminant) was present in 14 patients (48.3%) and had a mean latency of 84.4 days. Only four patients (13.8%) suffered from fulminant T1DM on initial admission, where it was its visceral manifestation. Acute pancreatitis was the second most frequent manifestation of GIT involvement in DS, affecting 11 patients (37.9%). The most common culprit medication was lamotrigine in three patients (27.3%). A complete list of the different modes of pancreatic involvement is demonstrated in Table 5.

#### 3.3.2. DRESS Colitis

We identified 21 patients (41.2%) in this review who developed colitis during DS. The most common symptoms of colitis were abdominal pain and diarrhea. A colon biopsy was performed in 13 out of the 21 patients (61.9%) who presented with colitis. The prototypical finding was inflammation and lymphocytic infiltration, mainly consisting of plasma cells and eosinophils. Four cases described the presence of ulcerations.

#### 3.3.3. DRESS Enteritis

The small bowel was affected in only three patients (5.9%) and manifested as non-bloody diarrhea. In all three cases, an upper GI endoscopy demonstrated changes in the duodenum.

#### 3.3.4. DRESS Esophagitis

This systematic review identified four patients (7.8%) with esophageal injury due to DS. Three patients presented with symptoms suggestive of esophageal involvement manifested by dysphagia and chest and/or epigastric pain, while one patient presented atypically with a distended abdomen. An esophagogastroduodenoscopy (EGD) in all three cases demonstrated macroscopic signs of inflammation. Two of these patients had white exudates/membranes, while one patient’s EGD demonstrated mucosal erosions. One case concurrently had esophageal candidiasis. An esophageal biopsy was performed in three cases. Two of these described significant mucosal eosinophilic infiltration that was not present in other parts of the alimentary tract (i.e., stomach and duodenum). The presence of CMV was excluded in one case.

#### 3.3.5. Gastric Involvement in DRESS Syndrome

The stomach was affected in only one patient, who presented with non-bloody diarrhea and protein-caloric malnutrition. Although the endoscopy was normal, a stomach biopsy demonstrated the loss of parietal cells, glandular disarray, inflammation and apoptosis. Further disease course included the development of autoimmune enteropathy as a sequela. This patient also had positive serology for HHV6 and CMV, but negative CMV immunohistochemical stains on the GIT biopsy.

### 3.4. Other Internal Organ Involvement in DRESS Syndrome

Consistent with previous reports, the most common visceral organ affected by DRESS in this cohort of patients was the liver in 38 patients (74.5%). Other internal organs were less frequently involved: kidney in 20 patients (39.2%), lungs in nine patients (17.6%), heart in six patients (11.8%) and thyroid gland in six patients as well (11.8%).

### 3.5. Viral Involvement

In this systematic review, we documented cases that demonstrated the presence of human herpesvirus 6 (HHV6), Epstein–Barr virus (EBV) and cytomegalovirus (CMV) reactivation. The total number of case reports that were tested for the presence of viral reactivation was 22 (43.1%) for HHV6, 21 (41.2%) for EBV and 23 (45.1%) for CMV. Twelve patients (23.5%) tested positive for HHV6. CMV and EBV were much less frequent, positive in only four (7.8%) and two patients (3.9%), respectively. Two patients tested positive for both HHV6 and CMV reactivation, and one patient for both HHV6 and EBV.

### 3.6. Therapy and Outcome

In our systematic review, the majority of patients, 39 (76.5%), received steroid treatment. Of the 51 patients, 43 (84.3%) survived and eight patients (15.7%) died due to DS complications. The pancreas was involved in six (75%) of the patients who died, followed by the colon in three (37.5%). A complete list of the treatment utilized is presented in Table 6.

## 4. Discussion

DS can occur in any age range, although prevalence is higher in the adult population, as was confirmed by other systematic reviews [10]. There is no specific sex predilection and both males and females are affected equally. The association between DRESS and specific HLA haplotypes has been established previously. For example, HLA-A31:01 is a risk factor for the development of all the severe cutaneous reactions to carbamazepine but its association with DS is particularly strong. Other haplotypes such as HLA-B*5701, HLA-A*3101, HLA-B*5801, HLA-DR3 and HLA-DQ2 have also been implicated in predisposing individuals to the development of other SCARs [58,59,60,61].

The pathogenesis behind DS is puzzling and remains unknown. It seems to be multifactorial, involving specific gene mutations and HLA haplotypes, immunologic mechanisms and herpesviruses reactivation [11]. A mutation on certain genes can lead to abnormal medication metabolism and the accumulation of toxic metabolites, which, in turn, might lead to an abnormal activation of the immune system. Certain individuals with immunological predisposition can become sensitized to these metabolites and respond by T cell activation, cytokine expansion and an increase in peripheral eosinophil counts [11]. Herpesvirus infection/reactivation is thought to predispose the affected individuals to develop an aberrant immunologic response as well. Among herpesviruses, the strongest supporting evidence exists for HHV6 involvement, which can usually be detected 2–4 weeks after symptom onset [6].

Eosinophils secrete highly toxic proteins that increase the production of free radicals, induce cell necrosis and apoptosis, which seems to be the reason for multiple organ damage in the syndromes [62]. Depending on the type of organ involvement, an increased absolute eosinophil count (AEC) may or may not be associated with disease severity. In cases of lung involvement, Taweesedt et al. did not demonstrate a significant association [10]. Contrary to this, the severity of myocarditis is often predisposed by a high AEC [62]. The prevalence of eosinophilia in patients with DS has been reported as high as 95% in previous reports [12].

Since DRESS occurs after adding new medication, it is critical to analyze its latency period. Previous reviews reported the mean latency of 27.3 days (3.9 weeks) [1]. In cases of re-challenge (either accidental or intentional), a significantly shorter latency period has been observed [11,63]. Our review has found the latency to be longer and one of the explanations behind this observation is that the autoimmune sequelae listed as GIT manifestations usually occur late in the disease course. Recently, Soria et al. postulated a possible association between certain drug classes and the latency of DS onset [3]. They found that antibiotics were a more common culprit in the group of patients with short latency (≤15 days) and anticonvulsants in the group with longer latency (>15 days). This observation was not evident in our systematic review.

The GIT seems to be affected to a lesser degree in DS than other internal organs. However, GIT involvement might also be underreported since many manifestations are non-specific, short lasting and usually do not warrant further investigation. In rare cases, GIT manifestations are dramatic and life threatening, as reported by Kano et al., who noted abrupt gastrointestinal bleeding caused by cytomegalovirus (CMV) gastric ulcerations that developed as a part of DS [6]. Ulceration as part of DS most commonly occurs in the large bowel and the association with CMV is variable.

Diarrhea was the most common presenting symptom in 43.1% of the patients. The incidence of bloody and non-bloody diarrhea was the same, and, in some cases, diarrhea was initially non-bloody only to turn to hematochezia later in the course of the disease. The cause of diarrhea was either colitis/enteritis or pancreatic failure, where it presented as steatorrhea due to an exocrine pancreatic insufficiency.

Based on the biopsy findings described in this review and the probable pathophysiologic mechanisms previously described, we postulate that there is an aberrant immunologic response that causes the infiltration of lymphocytes and/or eosinophils in lamina propria and submucosal parts of the digestive tract. These secrete pro-inflammatory molecules (e.g., cytokines), causing inflammation, which leads to epithelial injury and ulcerations. Furthermore, eosinophil degranulation in the tissue releases several components that directly lead to tissue damage [62]. Severe cases can present with complete bowel wall destruction and perforations, as illustrated in two case reports [20,21].

The most common manifestation of pancreas involvement was T1DM. Diagnosing DM in this setting can pose a challenge for a clinician as the frequent administration of steroids in DS with visceral involvement (either in oral or parenteral form) frequently leads to steroid-induced hyperglycemia. However, it is important to note that T1DM is more frequently a sequela rather that an acute manifestation of DS. 

The following two types of diabetes mellitus are generally described here: autoimmune and fulminant.

Autoimmune T1DM is characterized by the presence of antiglutamic acid decarboxylase (GADA) and islet cell (ICA) antibodies and is usually associated with other autoimmune manifestations such as hypothyroidism [6]. We found three reports of patients developing concomitant hypothyroidism and autoimmune DM. In some cases, other autoimmune disorders such as alopecia can develop concurrently with autoimmune DM as the late sequelae of DS [33,64]. The pathogenesis of this complication has not been fully elucidated; however, the dysfunction of regulatory T cells (Tregs) is thought to be a significant factor. Tregs are responsible for suppressing the immune system and providing tolerance to self-antigens, thereby preventing autoimmune diseases. In DS, the number of Tregs remains normal, but their function is altered, and they lose their ability to maintain tolerance to self-antigens, which results in the proliferation of effector T cells and cytokine release, consequently leading the immune system to fail at discriminating self-antigens from other antigens [57,65].

Unlike autoimmune T1DM, the fulminant form of the disease manifests during acute illness, characterized by a negative antibody profile (anti GAD, ICA), and presents as a ketoacidosis or hyperosmolar non-ketogenic state [6]. Fulminant T1DM as a sequela of DS occurs rarely, as early as 6 weeks after the other symptoms and signs of the syndrome are resolved, as illustrated in a case report where the patient returned to the hospital in diabetic ketoacidosis six weeks after the DS was successfully treated [14]. The patient in the aforementioned case report had documented HHV6 reactivation and the authors suggest that these autoimmune sequelae might be more frequent in patients who experience viral reactivation during the course of the disease. This fulminant form is usually non-autoimmune since autoimmune antibodies are absent [6,14,57].

T2DM is not typically associated with late complications of DS. Interestingly, one case report described a female pediatric patient, 14 years of age, who developed hyperglycemia on the second day of steroid treatment and was diagnosed with T2DM [5]. This diagnosis persisted at a one-year follow-up and necessitated the continued use of oral antidiabetics. The diagnosis (T2DM rather than T1DM) was confirmed by negative antibodies (ICA, GADA) and gene mutations for maturity onset diabetes of the young (MODY).

After the above-mentioned various types of diabetes, acute pancreatitis was the second most common mode of pancreatic involvement.

Acute pancreatitis due to DRESS likely has complex and multifactorial etiology. Rather than just simple viral reactivation, cytokine production and alteration in immunological response to different drug metabolites contribute to the development of pancreatitis in this patient population. Hence, it can be challenging to differentiate acute pancreatitis as part of DS, from those of viral etiology. It seems particularly difficult to distinguish DRESS pancreatitis associated with the reactivation of viruses known to cause acute pancreatitis such as cytomegalovirus (CMV) or Epstein–Barr virus (EBV) [17,50]. In cases of DRESS pancreatitis with documented HHV6 reactivations, it is somewhat easier as HHV6 was not documented to cause acute pancreatitis [7].

Following the primary infection, which usually occurs in early childhood, HHV6 establishes latency in salivary glands, lymphocytes and monocytes. During periods of immunosuppression, the virus can reactivate, causing encephalitis and pneumonitis, but pancreatitis has not been reported [7,66]. It is important to keep in mind another differential diagnosis—drug-induced acute pancreatitis (DIAP). Both can present with almost identical symptoms (i.e., abdominal pain, nausea, vomiting) and share the common culprits, such as carbamazepine, lamotrigine, sulfasalazine, dapsone and others [67]. Multiorgan failure can occur in both DS and as a complication of acute pancreatitis. Latency in DIAP ranges from one week to a month [68], while pancreatitis in cases of DRESS is somewhat longer, at 35.2 days (range of 2–90). Although elevated leukocytes can present in both, eosinophilia, atypical lymphocytosis, rash and lymphadenopathy are all absent in drug-induced pancreatitis.

Steroids are the mainstay of treatment for DRESS with visceral involvement (including pancreatitis). On the other hand, steroids are one of the medications traditionally associated with drug-induced pancreatitis [69]; therefore, making a clear distinction between these remains of the utmost importance as treatment might differ. Acute pancreatitis remains a less recognized visceral manifestation of DS, as illustrated by the fact that it went unrecognized in almost half of the patients included in this review and the fact that only 54.5% of those who presented with DRESS-induced pancreatitis received appropriate and timely steroid treatment.

A histological evaluation of the pancreas was performed in one patient included in our review [50]. This patient suffered from DRESS-induced fulminant T1DM and later died due to the development of heart failure and septicemia. An autopsy evaluation of the pancreatic tissue revealed many positive CMV cells in the alpha cells (glucagon-secreting) and exocrine parts of the pancreas. Significant inflammatory infiltration of CMV-positive islets was also revealed. The beta cell area (insulin-secreting) was decreased by 97% and no CMV cells were located in this region. The authors propose that beta cells could have been previously infected by CMV, which induced an immune response and activation of autoreactive T cells, leading to the death of beta cells and fulminant T1DM.

This review found the colon to be the second most common GIT organ affected in DRESS syndrome, after the pancreas. However, if pancreatic manifestations are subdivided on acute manifestation and late sequelae, then colitis would be the most common GIT manifestation of DS in this review (note that the liver was not analyzed in this review). While some patients are diagnosed with colitis based on a combination of diarrhea, abdominal pain and CT findings, others undergo a colonoscopy. Of the 21 patients with colitis, 13 (61.9%) underwent an endoscopy. Findings from the colonoscopies in these 13 patients yielded important results. Inflammation, mucosal ulcerations, hemorrhage, friable mucosa, white exudates and contact bleeding were among the most common macroscopic characteristics described. Additionally, some cases reported the infiltration of the bowel wall with eosinophils [39]. Similar to the pathophysiology of other visceral organs, where eosinophils cause the tissue damage, in bowels they might lead to damage of mucosa and submucosa, endothelitis, the formation of intravascular thrombi and ultimate bowel ischemia. Three patients had severe DRESS colitis, which, in one case, led to multiple perforations and pneumoperitoneum [20]. Although the patient in this case survived, an extensive colon resection with an ileostomy was necessary. In some cases, DRESS colitis can also present as a massive hemorrhage, leading to a fatal outcome [18]. One patient in our review had immunohistochemically confirmed CMV colitis [32]. This patient had an elevated CMV viral load 42 days after admission for DRESS and was treated with IV ganciclovir. The infection lasted for 18 days, but the patient continued to experience relapses of rash and eosinophilia after its resolution. Cases such as this one might support the theory of viral reactivation as part of complex DRESS pathogenesis.

Three patients presented with small bowel involvement. In two of these cases, the specific histopathology findings included duodenal inflammation, villous atrophy and the loss of Paneth and goblet cells. One case reported duodenal granulomas and micro-abscess formation. Interestingly, autoimmune enteropathy has also been described as an autoimmune sequela following DS diagnosis [8], where the patient presented with diarrhea that lasted for two months after the initial DRESS diagnoses. Biopsies of the duodenum revealed villous blunting and the absence of goblet and Paneth cells. This patient also tested positive for anti-enterocyte antibodies, further confirming the diagnosis. Although uncommon, autoimmune enteropathy is yet another autoimmune sequela in DRESS. In cases with significant GIT involvement, particularly if the small bowel and stomach are affected, the administration of steroids orally should be avoided and the parenteral form should be switched to as absorption might be inadequate. 

Regarding esophageal involvement in DS, an important differential diagnosis includes eosinophilic esophagitis (EoE). Unlike DRESS esophagitis, EoE is a chronic condition. Clinically, EoE presents similarly to DRESS esophagitis with dysphagia or odynophagia. Eosinophilic infiltration noticed on biopsy is similar to DRESS esophagitis [70], but for a diagnosis of EoE, a minimum of 15 eosinophils per high power microscopy field need to be detected and proton-pump inhibitor responsive esophageal eosinophilia (PPI-REE) needs to be excluded [71].

Clinicians can differentiate between these two conditions based on the timing of symptoms. EoE is a chronic lifelong diagnosis and spontaneous remission is rare, while DS is an acute disease with a clear temporal relation between drug exposure and symptom occurrence. The antigens involved in EoE pathophysiology seem to be predominantly food based; however, some antibiotics can contribute to its development, whereas DS is not caused by food antigens [70]. EoE usually warrants chronic anti-inflammatory therapy, unlike DS, where immunosuppressants are used temporarily. 

Two of the four patients included in this review were referred for further evaluation under suspicion of EoE, when in fact, esophagitis was part of their DS manifestation [54]. This exemplified the difficulties in differentiating these conditions and the need for raising awareness about these two conditions that are both rare and have similar clinical presentation. Sometimes the lack of esophageal symptoms does not lead to proper esophageal examination (e.g., EGD and biopsy) and these cases with asymptomatic esophageal involvement might contribute to under-diagnosis and under-reporting of esophagitis as a visceral manifestation of DS.

While the pathogenesis of DS remains unclear, many proposed theories have included viral reactivation as one of the most important events that might determine who develops DS. Viruses from the Herpesvirdiae family have been the most commonly associated with this syndrome. However, there is no consensus in regard to how common and/or crucial viral reactivation is for the development of the syndrome. In Europe and North America, testing for viral reactivation is not common clinical practice, while in Asia it is almost standard practice. These geographical differences are reflected in that a Japanese expert group has proposed J-SCAR criteria for the diagnosis of DS. While many criteria are similar to RegiSCAR, a notable exception is documented viral reactivation, which is included in the former. Depending on the evidence of HHV6 reactivation, cases might be defined as typical (if HHV6 reactivation is documented) or atypical in the cases where reactivation is absent [72]. Some authors hypothesized that patients with DS who demonstrated evidence of viral reactivation had more severe clinical manifestations and worse outcomes [3,73]. Additionally, viral reactivation might contribute to a prolonged course of the illness as well as relapses during steroid taper [38].

Treatment of DS has never been evaluated in prospective clinical trials. Our experience in its management is based on case reports, case series and retrospective studies. The rarity of the syndrome precludes more rigorous studies to be conducted.

In mild DS, where skin manifestations are predominant and there are no significant visceral organs involved, topical steroids, antihistamines and cessation of the culprit drug is enough [74].

Visceral organ involvement mandates the use of immunosuppressive therapy—most commonly steroids. In a case of steroid failure or contraindications, steroid sparing options have been used (cyclosporine, cyclophosphamide, intravenous immunoglobulins (IVIG) and plasma exchange). In this review, five patients received IVIG [4,15,40,49,51] and two patients received plasma exchange [4,52].

Cyclosporine, through its effect on interleukin 5 production (essential for the generation of eosinophilia), is particularly promising and an attractive option. A recent study [75], although limited by the small sample size, demonstrated that cyclosporine might be more effective than steroids in reducing DS progression. Furthermore, cyclosporine use has not been associated with the viral re-activation that might occur with steroids [75]. A recent case report documented treatment with the JAK kinase inhibitor tofacitinib in a patient who had refractory DS and was not responding to other immunosuppressive agents. Through advanced single-cell RNA sequencing from skin and blood tissue (sc RNA-seq), the authors demonstrated that the JAK-STAT signaling pathway was involved in the pathogenesis of DS. Following the treatment with tofacitinib, the patient improved and was weaned off other immunosuppressants [76].

Additionally, benralizumab (IL-5-receptor specific humanized monoclonal IgG antibody) was successfully used in the treatment of DS in two severely ill COVID-19 patients [77]. While some authors have reported concerns with the use of steroids (which, theoretically, could exacerbate viral reactivation due to the immunosuppressive effect), the fact that many patients respond favorably to steroids, and that relapses frequently occur following steroid taper, establish steroids as the main treatment for severe DS.

Our systematic review has few limitations. First, we have only included case reports and case series from journals indexed in PubMed. Consequently, we might have missed some high-quality reports in journals not indexed in this database. Secondly, we have not evaluated liver (previously extensively reported) and gallbladder involvement in DS.

## 5. Conclusions

DS can involve any part of the GIT and the liver is the most affected organ. Among the lesser-known gastrointestinal manifestations of DS, colitis, pancreatitis, autoimmune T1DM, fulminant T1DM and T2DM are the most common, sometimes as a late sequela. Rare, but equally important, are DRESS esophagitis, gastritis and enteritis. These rarely recognized and infrequent entities can manifest dramatically, and their development might be associated with increased mortality. With the exception of the liver, other gastrointestinal organs are rarely the sole visceral manifestations of DS, and they usually occur as part of multiorgan involvement. This might lead to higher mortality in this group of patients (15.7%) than previously reported for DS. The treatment of these complications follows the general rule for severe DS, which includes the use of immunosuppressants, most commonly steroids. In the case of severe GIT involvement, the oral route should be avoided due to unpredictable absorption and the parenteral route is preferred. Due to nonspecific and self-limiting signs and symptoms, gastrointestinal manifestations (apart from hepatitis) are frequently underrecognized, under investigated and underreported.

## Figures and Tables

**Table 1:** This table summarizes all co-morbid conditions of patients described in this systematic review. Patients who had no comorbidities were given the culprit drug for an acute disease (i.e., infection, pain, acne, etc.). Multiple patients had multiple comorbidities.

Chronic Co-Morbid Conditions	Number of Patients
None	11/51 (21.6%)
Epilepsy/seizure disorder	11/51 (21.6%)
Hypertension	7/51 (13.7%)
Hyperuricemia/Gout	5/51 (9.8%)
Bipolar disorder	4/51 (7.8%)
Thyroid disease	4/51 (7.8%)
Chronic kidney disease	4/51 (7.8%)
Diabetes/pre-diabetes	3/51 (5.9%)
Arrhythmia	2/51 (3.9%)
Psoriatic arthritis	2/51 (3.9%)
Other less common comorbidities: asthma, alcohol abuse, arrhythmia, chronic heart failure, chronic cough, coronary artery disease, duodenal ulcer, dyslipidemia, idiopathic peripheral neuropathy, mitral valve prolapse, multiple myeloma, multiple sclerosis, opiate use disorder, pulmonary hypertension, pemphigus, primary sclerosing cholangitis, psoriatic arthritis, rheumatoid arthritis, schizophrenia, severe pes excavatum, trigeminal neuralgia, ulcerative colitis and tuberculosis.	All other comorbidities listed were present in only one patient.

**Table 2:** This table lists the most common culprit medication in patients included in our systematic review. Italicized medications were suspected of being the culprit drug, but the exact cause was not confirmed.

Medication	Number of Cases
Carbamazepine	11/51 (21.6%)
Allopurinol	5/51 (9.8%)
Lamotrigine	5/51 (9.8%)
Dapsone	2/51 (3.9%)
Mexiletine	2/51 (3.9%)
Minocycline	2/51 (3.9%)
Sulfasalazine	2/51 (3.9%)
Vancomycin	2/51 (3.9%)
Amoxicillin–Clavulanate	1/51 (1.9%)
Ciprofloxacin	1/51 (1.9%)
Clindamycin	1/51 (1.9%)
Leflunomide	1/51 (1.9%)
Methicillin sodium	1/51 (1.9%)
Methimazole	1/51 (1.9%)
Naproxen	1/51 (1.9%)
Piperacillin–Tazobactam	1/51 (1.9%)
Phenytoin	1/51 (1.9%)
Phenobarbital	1/51 (1.9%)
Sodium Aurothiomalate (Gold)	1/51 (1.9%)
Spironolactone	1/51 (1.9%)
Valproic acid	1/51 (1.9%)
*Antituberculotics*	1/51 (1.9%)
*Titanium bioprothesis/Minocycline/Rifampicin*	1/51 (1.9%)
*Diclofenac/Ibuprofen*	1/51 (1.9%)
*Lamotrigine/Bupropion*	1/51 (1.9%)
*Phenytoin/Lamotrigine*	1/51 (1.9%)
*Chemotherapeutics (vincristine, doxorubicin, melphalan)*	1/51 (1.9%)
*Unspecified antibiotic*	1/51 (1.9%)

**Table 3:** This table summarizes most common gastrointestinal symptoms with which patients presented.

Signs/Symptoms		Number of Cases
Diarrhea *(n = 22)*	Unspecified type	10 (19.6%)
	Non-bloody	4 (7.8%)
	Bloody	4 (7.8%)
	Watery with positive occult blood	2 (3.9%)
	Non-bloody becoming bloody	2 (3.9%)
Vomiting		10/51 (19.6%)
Abdominal pain		8/51 (15.7%)
Nausea		7/51 (13.7%)
Epigastric pain/chest pain **		3/51 (5.9%)
Dysphagia		2/51 (3.9%)

** Presumably due to esophageal involvement.

**Table 4:** This table summarizes involvement of different component of alimentary tract.

Organ Affected	Number of Cases
Pancreas	29/51 (56.9%)
Colon	21/51 (41.2%)
Esophagus	4/51 (7.8%)
Small bowel	3/51 (5.9%)
Stomach	1/51 (1.9%)

**Table 5:** This table summarizes type of pancreas involvement, out of all patients who presented with pancreas involvement (*n* = 29) *.

Pancreas Involvement		Number of Cases
Acute pancreatitis		11/29 (37.9%)
T1DM 18/29 (62.1%)	Sequela: autoimmune T1DM	7/29 (24.1%)
	Sequela: fulminant T1DM	7/29 (24.1%)
	Admission: fulminant T1DM	4/29 (13.8%)
Sequela: type 2 diabetes mellitus		1/29 (3.4%)
Sequela: chronic pancreatic insufficiency		1/29 (3.4%)

* Multiple patients had more than one type of involvement.

**Table 6:** This table summarizes the treatment patients received during hospitalization.

Therapy Option	Number of Cases
Steroids (IV or PO)	39/51 (76.5%)
Antihistamines	5/51 (9.8%)
IVIG	5/51 (9.8%)
Ganciclovir	3/51 (5.9%)
Plasma exchange	2/51 (3.9%)

IVIG—intravenous immunoglobulins; IV—intravenous; PO—per os.

## Data Availability

Data are available from corresponding author upon request.
